# B-spline polynomials models for analyzing growth patterns of Guzerat young bulls in field performance tests

**DOI:** 10.5713/ab.23.0279

**Published:** 2024-01-20

**Authors:** Ricardo Costa Sousa, Fernando dos Santos Magaço, Daiane Cristina Becker Scalez, José Elivalto Guimarães Campelo, Clélia Soares de Assis, Idalmo Garcia Pereira

**Affiliations:** 1 Guzerat Agriculture and Livestock Selection LTDA, Rua Benjamin Constant, 366, Curvelo, MG 35790249, Brazil; 2 Department of Agriculture and Livestock, Faculty of Agricultural Science, Zambezi University, Ulónguè 2306, Mozambique; 3 Department of Animal Science, Faculty of Agricultural and Veterinary Sciences (FCAV), Jaboticabal, Universidade Estadual Paulista Júlio de Mesquita Filho – Unesp, SP 14884900, Brazil; 4 Department of Animal Science, Federal University of Piauí – UFPI, Teresina, Piauí 64049550, Brazil; 5 Department of Animal Science, Veterinary School, Federal University of Minas Gerais – UFMG, Belo Horizonte, MG 31270901, Brazil

**Keywords:** Allometry, B-Spline, Legendre, Random Regression, Zebu

## Abstract

**Objective:**

The aim of this study was to identify suitable polynomial regression for modeling the average growth trajectory and to estimate the relative development of the rib eye area, scrotal circumference, and morphometric measurements of Guzerat young bulls.

**Methods:**

A total of 45 recently weaned males, aged 325.8±28.0 days and weighing 219.9±38.05 kg, were evaluated. The animals were kept on *Brachiaria brizantha* pastures, received multiple supplementations, and were managed under uniform conditions for 294 days, with evaluations conducted every 56 days. The average growth trajectory was adjusted using ordinary polynomials, Legendre polynomials, and quadratic B-splines. The coefficient of determination, mean absolute deviation, mean square error, the value of the restricted likelihood function, Akaike information criteria, and consistent Akaike information criteria were applied to assess the quality of the fits. For the study of allometric growth, the power model was applied.

**Results:**

Ordinary polynomial and Legendre polynomial models of the fifth order provided the best fits. B-splines yielded the best fits in comparing models with the same number of parameters. Based on the restricted likelihood function, Akaike’s information criterion, and consistent Akaike’s information criterion, the B-splines model with six intervals described the growth trajectory of evaluated animals more smoothly and consistently. In the study of allometric growth, the evaluated traits exhibited negative heterogeneity (b<1) relative to the animals’ weight (p<0.01), indicating the precocity of Guzerat cattle for weight gain on pasture.

**Conclusion:**

Complementary studies of growth trajectory and allometry can help identify when an animal’s weight changes and thus assist in decision-making regarding management practices, nutritional requirements, and genetic selection strategies to optimize growth and animal performance.

## INTRODUCTION

Beef cattle herds in Brazil consist of various Zebu breeds due to their excellent maternal ability combined with their great adaptability to tropical environments [1]. Due to market demand for increased pasture-based meat production in tropical conditions, the Guzerat breed, originating from India [2], is one of the most commonly used in crossbreeding programs because of its adaptive traits [3].

The trend towards shorter production cycles and increased demand for quality meat, imply changes in the growth pattern to obtain early-maturing animals with great weight and finish. Thus, can meet through the study of growth curves that relate animal weight to age, by gathering a large number of measurements into a few parameters with biological interpretation [4]. Furthermore, grasping the intensity of growth could allow intervention in growth efficiency, management practices, and meat production [5].

The trend of increasing average body weight within different age groups measures the growth trajectory [4]. Estimating functions that describe animal growth is of particular interest in the use of random regression models in genetic evaluations of traits with repeated measurements over the animal’s lifetime [6]. These models draw on polynomials such as Legendre polynomials for instance [7–9], which can accurately describe the growth trajectory of animals. However, higher orders may lead to unreliable parameters at the beginning and end of the age interval, as they emphasize extreme observations [6]. An alternative to high-order Legendre polynomials is segmented B-spline polynomials, used to model the growth trajectories of beef cattle and the additive genetic and permanent environmental random effects [8,10]. In these models, each coefficient acts only on a fraction of the studied trajectory, providing better numerical properties and ease of parameter estimation [11].

Beyond the mathematical models that relate weight and age, the study of allometric growth complements these evaluations, allowing an understanding of the proportionality of tissue development in animals [5]. With Huxley’s allometric equation [12], the development of carcass tissues can be measured, determining the development pattern for economically important traits [5,13]. Since pasture-based performance tests (PPT) represent the country’s predominant beef cattle production system and aim to identify the best phenotypes as breeding stock, studying the growth of specific body parts related to the overall animal and their growth curves becomes crucial for such selection.

The goal of this study was to identify the suitable polynomial type and order for modeling the average growth trajectory and to evaluate morphometric meas Alongside the weighing, measurements of urements (hip height [HH], body length [BL], chest circumference [CC]), scrotal circumference (SC), and rib eye area (REA) (measured by ultrasound) relative to body weight of Guzerat bull calves in PPT.

## MATERIALS AND METHODS

### General information and database

Animal Care and Use Committee approval was not obtained for this study because no animal were handled in this experiment. The study was conducted using data from Guzerat cattle in a PPT carried out at Meleiro farm in the municipality of Curvelo, MG, Brazil, geographic located at 18°45′21” S - 44°25′51” O and altitude 672 m, Aw and Cwa, according to Köppen-Geiger (IBGE, 2019) climate classification, with precipitation of 1,094 mm. The PPT was coordinated by the Minas Gerais Zebu Breeders Association (AMCZ) and involved a master’s student from the Federal University of Jequitinhonha and Mucuri Valleys (UFJM). The animal evaluations followed the methodology of PPTs adopted by the Brazilian Zebu Breeders Association (ABCZ) and all welfare conditions were guaranteed.

A total of 45 recently weaned Guzerat bulls (Supplementary Table S1) representing the total number of animal that reflected actual field conditions were evaluated, with average initial ages and weights of 325.8±28.0 days and 219.9±38.05 kg, respectively. The animals were held under uniform management and feeding conditions for 294 days, grazing in *Brachiaria brizantha* pastures, and supplemented with protein salt. Additionally, the animals underwent three deworming treatments at intervals of 98 days during the test.

The animals were weighed at the beginning of the PPT on June 27, 2009, and after an adaptation period of 70 days, they were weighed again to initiate the performance test. Subsequent evaluations were conducted every 56 days (for a total of 224 days of testing) after a 12-hour fasting period.

Alongside the weighing, measurements of HH, BL, CC, SC, and REA using ultrasonography were taken. The measurements were made on the *Longissimus dorsi* muscle between the 12th and 13th ribs. These assessments were performed at each weighing, except during the adaptation period.

### Statistical analysis

Initial, data quality control analysis was performed. We evaluated the normality and homogeneity of residuals and data with potential measurement errors, which were not verified in the database.

The average growth trajectories were fitted using ordinary polynomials and the Legendre linear, quadratic, cubic, quartic, and quintic orders, as well as quadratic B-splines with two, three, four, five, and six regular intervals. The ages were standardized to the range from −1 to 1 to obtain the Legendre coefficients. The age standardization $\text{j}({\text{id}}_{\text{j}}^{*})$ was performed using the equation:


$${\text{id}}_{\text{j}}^{*}=\frac{2\left({\text{id}}_{\text{j}}-{\text{id}}_{\text{min}}\right)}{{\text{idmin}}_{\text{max}}-1},$$

where id_min_ and id_max_ represent the minimum and maximum ages of the range, respectively.

Next, the polynomial coefficients $\left(\phi_{\text{l}}({\text{id}}_{\text{j}}^{*})\right)$ were obtained using the equation [14]:


$$\left(\phi_{\text{l}}({\text{id}}_{\text{j}}^{*})\right)=\frac{1}{2^{\text{l}}}\sqrt{\frac{2\text{l}+1}{2}}\sum_{\text{m}=0}^{\left[\frac{1}{2}\right]}{\left(-1\right)}^{\text{m}}(1\;\text{m})\;(2\text{l}-2\text{m l}){\left({\text{id}}_{\text{j}}^{*}\right)}^{1-2\text{m}},$$

where, 1 represent the order of the polynomial, and the brackets [.] indicate rounding down to the nearest integer.

The coefficients of the quadratic B-spline functions were generated for the interval k, defined by the points T_k_ and T_k+1_, as follows: ϕ_k,0_(t_j_) = 1 and T_k_≤t_j_≤T_k+1_ or ϕ_k,0_ (t_j_) = 0, otherwise, where T_k_ represents the joining points of the segments or nodes. The coefficients of the B-spline functions of degree P were obtained using the following recursive function:


$$\phi_{\text{k},\text{P}}\left({\text{t}}_{\text{j}}\right)=\frac{{\text{t}}_{\text{j}}-{\text{T}}_{\text{k}}}{{\text{T}}_{\text{k}+\text{P}}-{\text{T}}_{\text{k}}}\phi_{\text{k},\text{P}-1}\left({\text{t}}_{\text{j}}\right)+\frac{{\text{T}}_{\text{k}+\text{P}+1}-{\text{t}}_{\text{j}}}{{\text{T}}_{\text{k}+\text{P}+1}-{\text{T}}_{\text{k}+1}}\phi_{\text{k}+1,\text{P}-1}\left({\text{t}}_{\text{j}}\right).$$

This study considered polynomial functions with up to six equidistant intervals. The division into m intervals required the specification of m–1 internal knots and two external knots (T_0_ and T_m_), resulting in m+1 knots and m+P non-null functions ϕ_(k,P)_. Additional p knots were added on each side of the interval [6].

The regression coefficients of each model were estimated using the restricted maximum likelihood method (REML). After estimating the regression coefficients and the weights at each age, the residuals were calculated for each observation to obtain the fit criteria. The following complementary criteria for model comparison based on the evaluation of the residuals of the fitted models: coefficient of determination (R^2^), mean absolute deviation (MAD), mean square error (MSE), and the value of the restricted likelihood function (–2RLL), Akaike’s information criterion (AIC), and consistent Akaike’s information criterion (CAIC) based on the number of parameters were considered (Table 1). Lower values of AIC and CAIC indicate better models. These criteria allow the comparison of non-hierarchical models and penalizing models with a higher number of parameters, with CAIC being more rigorous, thus favoring more parsimonious models.

The study of allometric (relative growth, of changes in proportion with increase in body weight) of the REA and morphometric measurements (HH, CC, BL, and SC) were conducted in relation to the body weight of the animals. The allometric growth was analyzed using the power model: $Y_{i}=aX_{i}^{b}{ɛ}_{i}$, where transformed logarithmically into a linear model [12]:


$$ln\;Y=ln\;a+bln\;X+ln\;{ɛ}_{i},$$

where: *Y* is the dependent variable (REA, HH, CC, BL, and SC); *X* is the body weight (in kg); *a* is the intercept of the logarithm of the linear regression on “*Y*” and “*b*”; *b* is the coefficient of relative growth or allometric coefficient; *ɛ_i_* is a multiplicative error, as it is the most applied model in allometric studies [15,16].

The *t*-test was applied at a significance level of 5% to test the hypothesis H_0_: b = 1. Growth was considered isogonic when b = 1, indicating similar development rates of “X” and “Y” in the evaluated growth interval. In the case of b ≠ 1, growth was considered heterogonic: when b>1 and positive, it reflects that “Y” developed proportionally more than “X”; when b<1 and negative, the intensity of “Y” development is lower than that of “X”. Data editing, consistency analysis, and model fit were performed using SAS version 15.1 software and the PROC REG procedure [17].

## RESULTS AND DISCUSSION

For the Guzerat young bulls (325.8±28.0 days), managed in the field during the offseason in the region, it is observed in association with the increase in weight and age of the animals, an increase in the evaluated morphometric traits (Table 2), but within the range observed in the literature for zebu cattle at the same age. For instance, REA, which is an indicator of the muscularity degree of the animal commonly used to assess the yield of higher commercial value meat cuts, shows a positive correlation with them [18], as well as with other important carcass components.

The SC during the PPT, ranging from 21 to 31 cm, differs from the results reported by Mamede et al [19] for the same age range in the Nelore breed (19.73 cm and 21.68 cm at 365 and 450 days of age, respectively). Compared to Guzerat breed animals, the values obtained were higher than the variation of 20.8 to 23.3 cm [20] and 20.87 to 23.24 cm [21].

The age at puberty can potentially influence SC. Considering the point of maximum inflection of SC at 13.2 months of age with 18.1 cm, as found by Osorio et al [22] who evaluated testicular development using a logistic function, the animals in the present study had already reached puberty, which supports the observed differences among several studies.

Field performance testing is a management strategy with the potential to identify genetically superior animals for meat quantity, carcass quality, and reproduction. According to [5], the factors that determine tissue growth and development in the body are crucial in breeding programs as they enable the adjustment of nutritional management, environmental conditions, and other aspects to shift the quantity and quality of meat produced.

One way to assess animal growth more flexibly is through growth trajectories, which allows monitoring of animal development, especially during the accelerated growth phase, to identify moments of changes [4]. This can be related to changes in the proportion of prime cuts of carcass or at the reproductive level, such as SC, which is seldom assessed in field performance test. In modeling the growth trajectory of Guzerat young bulls, it is noteworthy that the more parameterized models provided the best fit within each type of polynomial (Table 3). Ordinary and fifth-order Legendre polynomials showed the best fit, while among B-splines, those with five and six intervals had the best fits according to the MAD and MSE criteria, respectively (Table 3). Based on the R^2^, MAD, and MSE criteria, models with low errors were the most appropriate; however, they were also more complex.

Polynomials with the same number of parameters showed similar results according to R^2^. However, B-spline polynomials with two and three intervals showed a better fit compared to ordinary and cubic or quartic Legendre polynomials, respectively, according to the MAD and MSE criteria (Table 3). Despite differences in growth patterns among breeds, it is evident that higher-order models provided better fits. The results of the present study are consistent with Scalez et al [8], who observed better fits for Legendre and B-spline polynomials with higher orders in modeling weights of Nelore young bulls on PPT.

The linear ordinary polynomials, quintic Legendre polynomials, and B-spline polynomials with six intervals had the best fits according to the −2RLL and AIC criteria (Table 4). However, for the CAIC, variation was observed in the Legendre polynomial, where the quadratic polynomial had the best fit (Table 4). The AIC and CAIC criteria penalize more parameterized models for ordinary polynomials, making the linear ordinary polynomial the most appropriate when comparing within the same type of polynomial. However, the opposite was observed for Legendre and B-spline.

According to the −2RLL, AIC, and CAIC criteria for comparisons of all evaluated models, the B-spline polynomials provided better fits, with the model with six intervals being the best fit for the growth data of Guzerat young bulls (Table 3). Evaluating B-spline functions to model the growth of Nelore and Canchim cattle [23,24], respectively, reported the quadratic B-spline with four intervals as with best fit compared to Legendre functions, which is consistent with the results of this study. In another study, quadratic B-spline polynomials with three and four intervals were the best fit for modeling the growth of Nelore cattle and MA (21/32 Charolais +11/32 Nelore) cattle subjected to PPT [8].

**Figure 1:**
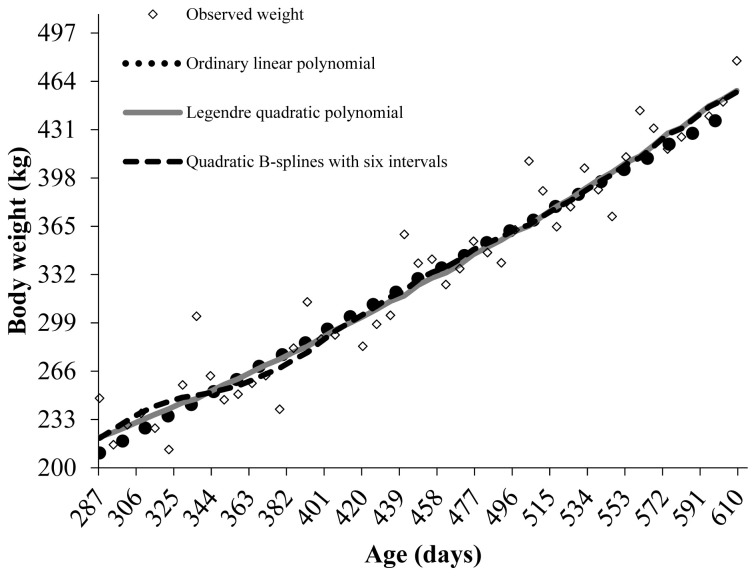
Growth trajectories of young Guzerat bulls in the performance tests of the evaluated tree models.

The linear ordinary polynomial showed significant oscillation at the edges of the age range considered (Figure 1). The B-spline with six intervals described the growth trajectory of Guzerat young bulls more smoothly and consistently, especially at the edges of the age range, with little difference from the quadratic Legendre polynomial.

The weights estimated by the linear ordinary polynomial after 553 days of age decreased, not reflecting the expected behavior for body weight. The quadratic Legendre polynomial and the B-spline with six intervals fit the data well, with their estimated weights after 483 days being close to each other and to the observed value (Figure 1).

The Legendre models with high orders may lead to inadequate parameter estimation as they emphasize observations at the edges associated with high computational cost [6]. These problems are mainly observed when the data sets contain a small number of records for the last ages, associated with a small number of records per animal [10], underpinning the observed results. In this sense, B-spline polynomials are more suitable for describing the growth trajectory of Guzerat young bulls in the field, as they offer better numerical properties, an easier parameter estimation process and are less susceptible to the problems frequently observed with orthogonal Legendre polynomials [11].

The application of splines as a way to represent animal growth is still recent, but different studies have reported their use in animal growth and genetic evaluation studies [6,8,23]. However, despite their great utility in practical situations, no studies on allometric growth have been conducted yet.

Therefore, through the Huxley equation [12] in the allometric growth analysis, it was observed that all variables showed heterogeneous growth (p<0.01) and allometric coefficients less than 1, showed differentiated growth in relation to body weight in the studied period, pointing precocity in these traits (Table 4).

The HH showed a lower coefficient b value compared to other traits, indicating that the animals grew in height earlier but continued to grow in BL, CC, SC, and REA. Similarly, the development of CC was proportionally greater (p<0.01) than body weight (Table 5). This demonstrates that the Guzerat breed has good rib curvature, allowing for a high feed intake capacity. Therefore, increased rib curvature is highly desirable as long as it does not compromise the overall body harmony of the animal.

Larger testicles provide a quantitative and qualitative increase in sperm production, leading to greater serving capacity and better libido in bulls [20,25]. The SC of the animals in the trial showed early development (p<0.01), as observed in Table 4, indicating that the young bulls will become reproductively mature early [22], which is desirable for the production system.

The SC has a favorable genetic correlation with age at first calving in females, as reported by [25,26], indicating the sexual precocity of the Guzerat breed. Selection for SC can genetically improve the precocity of the herd as it has moderate to high heritability estimates, providing a quick financial return to the producer [19].

The REA showed early development (Table 5), which is desirable in a cattle herd as it is moderately correlated with the animal’s weight gain [18], demonstrating high-value cuts and accumulated productivity [19], and that the growth of the *Longissimus dorsi* muscle is a good parameter for evaluating animal growth [16]. The allometric function used to describe allometric growth through the growth curves of body parts in relation to the body weight, by detecting high correlations between the traits [4], provides consistency to these statements.

Although the animals were contemporaries, the R^2^ values for the allometric growth study were moderate, probably due to the variation in their weights when entering the trial. This weight variation can be attributed to the farm effect, as the calves came from different breeders and could have been under different management conditions, especially regarding feeding.

However, considering that in performance tests the farm effect is controlled by keeping the test animals under the same environmental conditions so that only the differences between them represent additive genetic differences, the negative heterogony of the coefficients of the Huxley equation [12] was significantly evident, allowing the body structure to be linked to the function. Among the biometric measurements, CC and BL has been used to predict body weight due to the high correlation between them [27]. Significant correlations (p<0.01) were observed among biometric measurements, body volume, weight gain, body weight, and carcass fat thickness [28].

**Figure 2:**
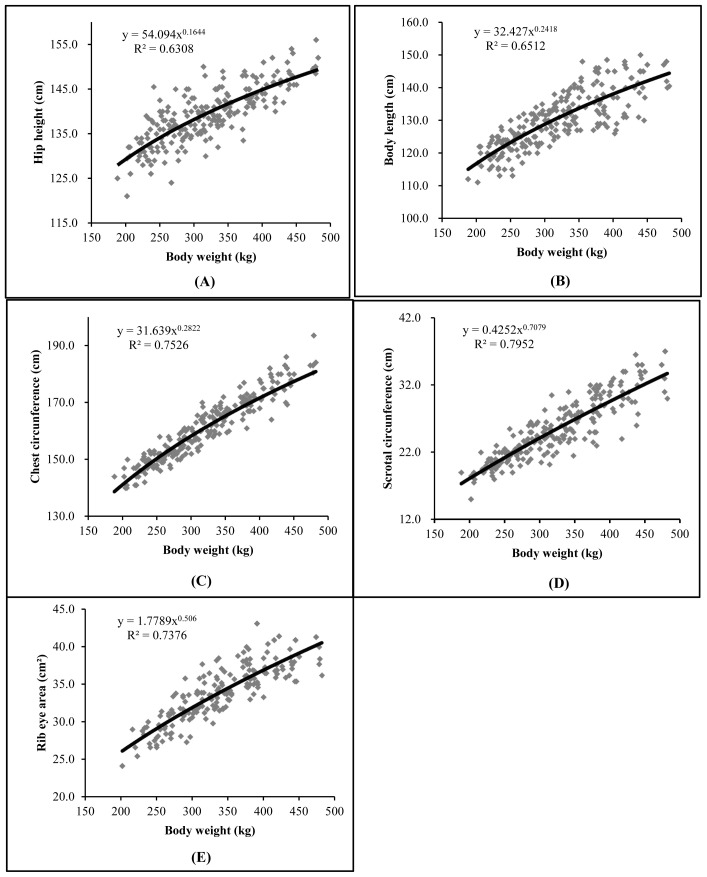
Allometric growth of hip height (A), body length (B), chest circumference (C), scrotal circumference (D), and rib eye area (E) in relation to body weight of Guzerat young bulls. The continuous black line represents the fitted model and the gray squares represent the observed values; R^2^, coefficient of determination.

Figure 2 presents the allometric equations and their respective regression curves for the variables evaluated regarding their development in relation to body weight. As previously mentioned, there is a reasonable dispersion of observed values. It can also be observed that the curves rise more steeply for all variables, exhibiting the precocity of the traits evaluated in Guzerat young bulls on pasture in the performance test.

This precocity demonstrated in the evaluated traits points those animals have developed a body structure that provides promising carcass capacity for meat deposition (muscle). Which is necessary for finishing in feedlots and pasture, strategies adopted to maximize production and adapt carcasses to the demands of the consumer market [29], as well as the potential for early reproduction based on SC. According to our findings, scientific literature lacks studies that complement the growth trajectories and relative growth of carcass components and reproductive organs in field performance tests. Given the observed sampling constraints in our study, future research should encompass varied data structures, breeds, and environmental conditions for a more holistic understanding.

## CONCLUSION

The application of higher-degree B-spline segmented polynomials proved to be a viable alternative for evaluating the growth trajectory of Guzerat bulls in field performance tests.

The evaluation of allometry displayed early maturation, pointing to valuable tools for selection applications in both higher-quality carcass cuts and reproduction in this breed.

Complementary studies of growth trajectory and allometry can help identify moments when an animal’s weight changes, and thus assist in decision-making regarding management practices, nutritional requirements, and genetic selection strategies to optimize growth and animal performance.

## Figures and Tables

**Table 1:** Criteria for model comparison

Criteria	Estimator
Mean absolute deviation	$\frac{1}{\text{n}}\sum_{\text{i}=1}^{\text{n}}\left|{\text{y}}_{\text{i}}-{\hat{\text{y}}}_{\text{i}}\right|$
Mean square error	$\frac{1}{\text{n}}\sum_{\text{i}=\text{i}}^{\text{n}}{\hat{\text{e}}}_{\text{i}}^{2}$
Value of the restricted likelihood function	(n−p) ln(2π) + ln|V|+ ln|X′V^−1^X| +(y−Xβ)′V^−1^(y−Xβ)
Akaike's information criterion	−2Ln(L)_x_ + 2(p_x_+k_x_)
Consistent Akaike's information criterion	−2Ln(L)_x_+(p_x_+k_x_) [Ln(n)+1]

n, represent the number of observations; y_i_ and ŷ_i_ the observed and estimated values, respectively; ê_i_^2^, estimated errors associated with each observation; p, represents the rank of the fixed effects matrix X, V is the covariance matrix of y_i_ (which represents the vector of observations); β, is the vector of fixed effects, and k is the number of model parameters.

**Table 2:** Means and standard deviations of the variables and animal body weight per evaluation

Trait	Assessment of animals during the trial (days)				

	0 (1st)	56 (2nd)	112 (3rd)	168 (4th)	224 (5th)
Body weight (kg)	248±32	269±34	320±39	360±41	406±44
Hip height (cm)	133±4	139±6	138±5	142±5	145±5
Body length (cm)	123±5	125±6	129±5	139±6	135±7
Chest circumference (cm)	151±6	153±6	160±7	168±7	171±12
Scrotal circumference (cm)	21±2	23±2	26±3	27±3	31±3
Rib eye area (cm^2^)	-	30±3	34±3	35±3	37±3

**Table 3:** Fit statistics for ordinary polynomials, Legendre polynomials, and quadratic B-splines for the average growth trajectory of Guzerat young bulls in a performance test

Model	NP	R^2^	MAD (kg)	MSE (kg^2^)
Ordinary polynomials				
Linear	2	0.9907	25.8117	993.2923
Quadratic	3	0.9909	25.4907	972.0300
Cubic	4	0.9910	25.4506	969.7300
Quartic	5	0.9910	25.3374	967.1800
Quintic	6	0.9910	25.3304	966.0100
Legendre polynomials				
Linear	2	0.9907	25.8117	993.2923
Quadratic	3	0.9909	25.4907	972.0300
Cubic	4	0.9910	25.4506	969.7300
Quartic	5	0.9910	25.3374	967.1800
Quintic	6	0.9910	25.3304	966.0100
Quadratic B-splines				
2 intervals	4	0.9910	25.4429	969.3300
3 intervals	5	0.9910	25.3218	966.2400
4 intervals	6	0.9910	25.3429	966.1500
5 intervals	7	0.9910	25.2981	962.9800
6 intervals	8	0.9910	25.3058	962.5800

NP, number of parameters; R^2^, coefficient of determination; MAD, mean absolute deviation; MSE, mean square error.

**Table 4:** Comparative criteria assessment for fitted polynomial models in analyzing the average growth trajectory of Guzerat young bulls in a performance test

Model	NP	−2RLL	AIC	CAIC
Ordinary polynomials				
Linear	2	2,193.5	2,199.5	2,212.748
Quadratic	3	2,203.0	2,211.0	2,228.664
Cubic	4	2,225.7	2,235.7	2,257.781
Quartic	5	2,257.2	2,269.2	2,295.697
Quintic	6	2,297.7	2,311.7	2,342.613
Legendre polynomials				
Linear	2	2,183.3	2,189.3	2,202.548
Quadratic	3	2,173.3	2,181.3	2,198.964
Cubic	4	2,167.4	2,177.4	2,199.481
Quartic	5	2,161.2	2,173.2	2,199.697
Quintic	6	2,154.9	2,168.9	2,199.813
Quadratic B-splines				
2 intervals	4	2,159.9	2,169.9	2,191.981
3 intervals	5	2,152.4	2,164.4	2,190.897
4 intervals	6	2,145.4	2,159.4	2,190.313
5 intervals	7	2,137.3	2,153.3	2,188.629
6 intervals	8	2,130.0	2,148.0	2,187.745

NP, number of parameters; −2RLL, restricted log-likelihood function; AIC, Akaike information criterion; CAIC, Consistent Akaike information criterion.

**Table 5:** Allometric coefficients in relation to body weight for morphometric measurements, scrotal circumference, and rib eye area of Guzerat young bulls

Trait	a	b	s(b)	R^2^	p-value
Hip height	54.0938	0.1644	0.0084	0.6308	**
Body length	32.4266	0.2418	0.0119	0.6512	**
Chest circumference	31.6393	0.2822	0.0108	0.7526	**
Scrotal circumference	0.4252	0.7079	0.0241	0.7952	**
Rib eye area	1.7789	0.5060	0.0226	0.7376	**

a, intercept of the linear regression; b, allometric coefficient; s(b), standard error of the allometric coefficient; R^2^, coefficient of determination.

** p<0.01.

## SUPPLEMENTARY MATERIAL

**Supplementary Table S1.** Field performance test data for young Guzerat bulls
