# Supplementary material for: B-spline polynomials models for analyzing growth patterns of Guzerat young bulls in field performance tests
Source: Anim Biosci. 2024 Jan 20;37(5):817–25. doi: 10.5713/ab.23.0279 (PMC11065712; doi:10.5713/ab.23.0279)
Supplement: Supplementary file 1 [file ab-23-0279-Supplementary-Table-1.pdf]

Supplementary table S1: Field performance test data for young Guzerat bulls

Region	Prod.	B.Date	In.weight	In. age	weight date	weight	age	HH	BL	CC	SC	REA
MAIA	1816	30/08/2008	245	301	05/09/2009	255	371	132.0	123.0	152.0	22.0	
MAIA	1816	30/08/2008	245	301	31/10/2009	283	427	133.5	132.0	157.0	24.0	31.8
MAIA	1816	30/08/2008	245	301	22/12/2009	334	479	137.0	133.0	164.0	28.5	38.2
MAIA	1816	30/08/2008	245	301	20/02/2010	379	539	143.0	146.0	171.0	30.0	39.7
MAIA	1816	30/08/2008	245	301	17/04/2010	422	595	149.0	134.5	173.0	34.0	41.4
CNS	7615	25/08/2008	245	306	05/09/2009	246	376	132.0	123.0	152.0	22.0	
CNS	7615	25/08/2008	245	306	31/10/2009	286	432	143.5	125.0	154.0	25.0	30.3
CNS	7615	25/08/2008	245	306	22/12/2009	359	484	139.5	134.0	163.0	28.0	35.7
CNS	7615	25/08/2008	245	306	20/02/2010	418	544	142.0	140.0	175.0	31.0	37.6
CNS	7615	25/08/2008	245	306	17/04/2010	450	600	146.0	147.0	180.0	34.0	38.7
CBF	1770	30/08/2008	318	301	05/09/2009	309	371	139.0	126.0	165.5	26.5	
CBF	1770	30/08/2008	318	301	31/10/2009	342	427	149.0	131.0	169.0	28.0	34.9
CBF	1770	30/08/2008	318	301	22/12/2009	381	479	145.0	129.0	173.0	32.0	38.4
CBF	1770	30/08/2008	318	301	20/02/2010	427	539	147.0	143.0	180.0	31.0	37.0
CBF	1770	30/08/2008	318	301	17/04/2010	474	595	149.5	147.0	183.0	35.0	41.3
FMN	696	19/08/2008	324	312	05/09/2009	314	382	139.0	134.0	164.0	23.0	
FMN	696	19/08/2008	324	312	31/10/2009	332	438	145.0	137.0	158.0	24.5	32.0
FMN	696	19/08/2008	324	312	22/12/2009	385	490	143.5	138.0	167.0	27.5	36.9
FMN	696	19/08/2008	324	312	20/02/2010	440	550	146.0	145.0	182.0	27.7	37.6
FMN	696	19/08/2008	324	312	17/04/2010	478	606	148.5	142.0	180.5	31.0	40.0
MVB	875	30/08/2008	262	301	05/09/2009	279	371	135.0	123.0	152.0	23.0	
MVB	875	30/08/2008	262	301	31/10/2009	313	427	140.0	124.0	160.0	23.0	33.9
MVB	875	30/08/2008	262	301	22/12/2009	387	479	142.0	132.0	169.0	29.0	36.8
MVB	875	30/08/2008	262	301	20/02/2010	426	539	146.0	143.0	177.0	30.0	37.4
MVB	875	30/08/2008	262	301	17/04/2010	478	595	150.0	148.0	183.0	33.0	37.7
GZR	90	30/08/2008	215	301	05/09/2009	225	371	128.0	119.0	146.0	20.5	
GZR	90	30/08/2008	215	301	31/10/2009	238	427	138.0	120.0	150.0	23.0	30.0
GZR	90	30/08/2008	215	301	22/12/2009	288	479	134.5	130.5	157.0	25.0	30.8
GZR	90	30/08/2008	215	301	20/02/2010	343	539	138.0	141.0	168.0	25.5	31.7
GZR	90	30/08/2008	215	301	17/04/2010	387	595	141.0	129.0	170.0	30.0	35.1
MAIA	1824	28/09/2008	252	272	05/09/2009	252	342	134.0	121.0	152.0	22.0	
MAIA	1824	28/09/2008	252	272	31/10/2009	273	398	145.0	124.0	151.0	22.0	31.2
MAIA	1824	28/09/2008	252	272	22/12/2009	334	450	142.5	126.0	160.0	25.9	36.0
MAIA	1824	28/09/2008	252	272	20/02/2010	364	510	140.0	137.0	169.0	24.0	38.8
MAIA	1824	28/09/2008	252	272	17/04/2010	406	566	146.0	137.0	175.0	28.0	40.4
CNS	7650	28/09/2008	276	272	05/09/2009	276	342	131.0	124.0	153.0	21.0	
CNS	7650	28/09/2008	276	272	31/10/2009	287	398	140.0	131.0	151.0	21.0	31.7
CNS	7650	28/09/2008	276	272	22/12/2009	341	450	137.0	130.0	159.0	23.5	35.2
CNS	7650	28/09/2008	276	272	20/02/2010	376	510	145.0	145.0	170.0	23.5	36.2
CNS	7650	28/09/2008	276	272	17/04/2010	438	566	145.0	138.5	170.0	26.0	38.6
DYP	89	19/09/2008	237	281	05/09/2009	231	351	135.0	118.0	151.0	19.5	
DYP	89	19/09/2008	237	281	31/10/2009	250	407	140.0	121.0	150.0	22.5	27.9
DYP	89	19/09/2008	237	281	22/12/2009	295	459	137.0	123.0	157.0	24.0	33.6
DYP	89	19/09/2008	237	281	20/02/2010	337	519	142.0	133.0	167.0	25.5	34.0
DYP	89	19/09/2008	237	281	17/04/2010	399	575	148.0	140.0	129.0	30.5	36.8
DYP	94	27/09/2008	260	273	05/09/2009	283	343	136.0	129.0	159.0	20.5	
DYP	94	27/09/2008	260	273	31/10/2009	319	399	141.0	125.0	163.0	21.5	30.4
DYP	94	27/09/2008	260	273	22/12/2009	377	451	143.5	129.0	171.0	26.5	35.1
DYP	94	27/09/2008	260	273	20/02/2010	417	511	148.0	147.0	179.0	24.0	35.9
DYP	94	27/09/2008	260	273	17/04/2010	482	567	152.0	140.5	184.0	30.0	36.2
DYP	97	30/09/2008	247	270	05/09/2009	229	340	130.0	123.0	150.0	21.0	
DYP	97	30/09/2008	247	270	31/10/2009	262	396	141.0	131.0	155.0	24.5	29.4
DYP	97	30/09/2008	247	270	22/12/2009	307	448	138.0	129.0	157.0	28.3	33.7
DYP	97	30/09/2008	247	270	20/02/2010	353	508	140.0	143.0	167.0	27.5	36.3
DYP	97	30/09/2008	247	270	17/04/2010	402	564	142.5	126.0	171.0	32.5	36.5
MVB	877	14/09/2008	271	286	05/09/2009	267	356	138.0	117.0	158.0	21.5	
MVB	877	14/09/2008	271	286	31/10/2009	289	412	145.0	120.0	155.0	24.0	31.1
MVB	877	14/09/2008	271	286	22/12/2009	337	464	145.5	128.5	162.0	27.6	38.5
MVB	877	14/09/2008	271	286	20/02/2010	376	524	145.0	137.0	172.0	28.0	40.0
MVB	877	14/09/2008	271	286	17/04/2010	415	580	152.0	131.0	182.0	32.0	40.9
MVB	879	16/09/2008	246	284	05/09/2009	254	354	140.0	126.0	158.0	22.0	
MVB	879	16/09/2008	246	284	31/10/2009	290	410	139.0	128.0	161.0	26.0	35.8
MVB	879	16/09/2008	246	284	22/12/2009	341	462	145.5	135.0	166.0	31.0	37.1
MVB	879	16/09/2008	246	284	20/02/2010	371	522	148.0	141.0	175.5	31.0	38.1
MVB	879	16/09/2008	246	284	17/04/2010	437	578	151.0	131.0	183.0	36.5	39.7

OMFJ	323	18/09/2008	326	282	05/09/2009	317	352	141.0	129.0	163.0	23.0	
OMFJ	323	18/09/2008	326	282	31/10/2009	331	408	145.0	136.0	165.0	26.0	31.7
OMFJ	323	18/09/2008	326	282	22/12/2009	386	460	148.0	134.0	172.0	32.0	33.7
OMFJ	323	18/09/2008	326	282	20/02/2010	439	520	149.0	145.0	186.0	32.0	38.0
OMFJ	323	18/09/2008	326	282	17/04/2010	479	576	156.0	140.0	193.5	37.0	38.4
GZR	93	20/09/2008	227	280	05/09/2009	239	350	131.0	124.0	153.0	20.0	
GZR	93	20/09/2008	227	280	31/10/2009	267	406	133.0	123.0	154.0	22.0	31.1
GZR	93	20/09/2008	227	280	22/12/2009	306	458	135.0	133.0	158.0	25.7	33.8
GZR	93	20/09/2008	227	280	20/02/2010	337	518	139.0	132.0	166.5	28.7	34.5
GZR	93	20/09/2008	227	280	17/04/2010	377	574	141.0	128.0	177.0	31.5	37.7
GZR	96	20/09/2008	271	280	05/09/2009	265	350	136.0	125.0	153.0	22.5	
GZR	96	20/09/2008	271	280	31/10/2009	287	406	136.0	123.0	154.0	22.5	33.5
GZR	96	20/09/2008	271	280	22/12/2009	345	458	142.5	129.0	162.0	25.4	36.7
GZR	96	20/09/2008	271	280	20/02/2010	374	518	149.0	145.0	170.0	27.0	36.1
GZR	96	20/09/2008	271	280	17/04/2010	416	574	146.5	132.0	180.0	32.5	36.8
MAIA	1828	25/10/2008	229	245	05/09/2009	227	315	131.0	120.0	146.0	21.0	
MAIA	1828	25/10/2008	229	245	31/10/2009	247	371	131.0	118.0	148.0	21.0	27.5
MAIA	1828	25/10/2008	229	245	22/12/2009	295	423	134.0	127.0	154.0	26.2	31.2
MAIA	1828	25/10/2008	229	245	20/02/2010	335	483	138.0	134.0	164.0	26.0	35.7
MAIA	1828	25/10/2008	229	245	17/04/2010	375	539	139.5	146.0	164.0	30.0	38.0
DYP	99	12/10/2008	273	258	05/09/2009	282	328	132.0	129.0	153.0	22.0	
DYP	99	12/10/2008	273	258	31/10/2009	301	384	136.0	130.0	161.0	23.5	31.7
DYP	99	12/10/2008	273	258	22/12/2009	335	436	136.5	133.0	159.0	26.0	33.2
DYP	99	12/10/2008	273	258	20/02/2010	353	496	142.0	142.0	166.5	26.5	34.2
DYP	99	12/10/2008	273	258	17/04/2010	419	552	143.0	146.0	171.0	30.0	36.9
BYDU	122	17/10/2008	204	253	05/09/2009	188	323	125.0	112.0	144.0	19.0	
BYDU	122	17/10/2008	204	253	31/10/2009	216	379	134.0	120.0	147.0	22.0	29.0
BYDU	122	17/10/2008	204	253	22/12/2009	257	431	128.5	125.0	149.0	25.5	31.4
BYDU	122	17/10/2008	204	253	20/02/2010	287	491	131.0	134.0	160.0	26.5	33.3
BYDU	122	17/10/2008	204	253	17/04/2010	317	547	133.5	133.0	164.5	30.5	35.2
GORI	198	20/10/2008	215	250	05/09/2009	237	320	128.0	123.0	148.0	19.5	
GORI	198	20/10/2008	215	250	31/10/2009	258	376	138.0	125.0	150.0	21.5	27.4
GORI	198	20/10/2008	215	250	22/12/2009	314	428	137.0	135.0	155.0	20.2	33.0
GORI	198	20/10/2008	215	250	20/02/2010	348	488	144.0	140.0	166.5	21.5	33.9
GORI	198	20/10/2008	215	250	17/04/2010	380	544	145.0	132.0	169.0	25.0	36.9
FMN	734	09/10/2008	292	261	05/09/2009	283	331	136.0	130.0	155.0	23.5	
FMN	734	09/10/2008	292	261	31/10/2009	297	387	143.0	133.0	157.0	26.5	28.0
FMN	734	09/10/2008	292	261	22/12/2009	350	439	139.5	137.0	165.0	27.5	33.0
FMN	734	09/10/2008	292	261	20/02/2010	400	499	144.0	140.0	178.0	31.0	33.3
FMN	734	09/10/2008	292	261	17/04/2010	446	555	146.0	137.0	178.0	35.0	35.4
MAFM	863	06/10/2008	305	264	05/09/2009	296	334	140.0	132.0	161.0	24.0	
MAFM	863	06/10/2008	305	264	31/10/2009	316	390	148.0	134.0	165.0	26.0	36.1
MAFM	863	06/10/2008	305	264	22/12/2009	380	442	145.0	135.0	170.0	30.5	37.7
MAFM	863	06/10/2008	305	264	20/02/2010	402	502	151.0	145.0	177.0	32.0	37.3
MAFM	863	06/10/2008	305	264	17/04/2010	443	558	154.0	140.0	180.0	34.0	38.0
ZOOA	129	07/10/2008	329	263	05/09/2009	311	333	141.0	133.0	157.0	22.0	
ZOOA	129	07/10/2008	329	263	31/10/2009	314	389	150.0	138.0	160.0	23.0	37.7
ZOOA	129	07/10/2008	329	263	22/12/2009	376	441	148.0	133.0	169.0	27.0	38.3
ZOOA	129	07/10/2008	329	263	20/02/2010	417	501	146.5	148.0	164.0	29.0	38.5
ZOOA	129	07/10/2008	329	263	17/04/2010	445	557	153.0	130.0	176.0	33.0	40.9
ZOOA	133	11/10/2008	211	259	05/09/2009	218	329	129.0	122.0	141.0	19.5	
ZOOA	133	11/10/2008	211	259	31/10/2009	250	385	136.0	130.0	147.0	22.5	26.9
ZOOA	133	11/10/2008	211	259	22/12/2009	305	437	135.0	130.0	157.0	25.0	31.6
ZOOA	133	11/10/2008	211	259	20/02/2010	338	497	138.0	140.0	166.0	25.2	31.5
ZOOA	133	11/10/2008	211	259	17/04/2010	386	553	141.5	133.0	167.0	30.5	35.0
ZOOA	134	11/10/2008	202	259	05/09/2009	220	329	130.0	118.0	144.0	19.5	
ZOOA	134	11/10/2008	202	259	31/10/2009	236	385	136.0	128.0	147.0	22.0	29.3
ZOOA	134	11/10/2008	202	259	22/12/2009	290	437	135.0	122.0	158.0	26.0	32.7
ZOOA	134	11/10/2008	202	259	20/02/2010	331	497	138.0	131.0	167.0	26.0	34.9
ZOOA	134	11/10/2008	202	259	17/04/2010	387	553	141.0	134.2	170.5	29.0	35.8
ZOOA	135	12/10/2008	292	258	05/09/2009	280	328	136.0	132.0	150.0	22.0	
ZOOA	135	12/10/2008	292	258	31/10/2009	292	384	145.0	132.0	152.0	23.5	27.3
ZOOA	135	12/10/2008	292	258	22/12/2009	352	436	141.0	140.0	159.5	29.5	32.9
ZOOA	135	12/10/2008	292	258	20/02/2010	391	496	144.0	148.5	167.0	31.0	35.3
ZOOA	135	12/10/2008	292	258	17/04/2010	440	552	148.5	150.0	169.3	35.0	37.1
IVAG	654	31/10/2008	188	239	05/09/2009	207	309	126.0	116.0	150.0	18.0	
IVAG	654	31/10/2008	188	239	31/10/2009	223	365	134.0	116.0	148.0	20.0	25.4
IVAG	654	31/10/2008	188	239	22/12/2009	271	417	134.5	120.0	153.0	22.9	31.6

IVAG	654	31/10/2008	188	239	20/02/2010	317	477	130.0	135.5	168.5	24.5	31.2
IVAG	654	31/10/2008	188	239	17/04/2010	347	533	141.0	127.0	172.0	29.0	32.0
GFCZ	8	25/11/2008	230	214	05/09/2009	234	284	129.0	113.0	149.0	20.0	
GFCZ	8	25/11/2008	230	214	31/10/2009	253	340	133.0	113.0	152.0	20.5	29.6
GFCZ	8	25/11/2008	230	214	22/12/2009	310	392	135.0	128.0	153.0	24.3	35.8
GFCZ	8	25/11/2008	230	214	20/02/2010	327	452	138.0	133.0	165.0	27.0	33.5
GFCZ	8	25/11/2008	230	214	17/04/2010	381	508	142.0	136.0	161.0	31.0	35.0
GORI	205	14/11/2008	200	225	05/09/2009	206	295	132.0	122.0	141.0	17.5	
GORI	205	14/11/2008	200	225	31/10/2009	230	351	139.0	125.0	146.0	20.0	28.8
GORI	205	14/11/2008	200	225	22/12/2009	267	403	136.0	117.0	152.0	22.5	30.8
GORI	205	14/11/2008	200	225	20/02/2010	301	463	137.0	131.5	156.0	23.5	31.1
GORI	205	14/11/2008	200	225	17/04/2010	329	519	141.0	127.0	160.0	28.5	32.2
MNCG	75	17/11/2008	204	222	05/09/2009	202	292	121.0	111.0	147.0	15.0	
MNCG	75	17/11/2008	204	222	31/10/2009	232	348	129.0	115.0	142.0	19.0	28.3
MNCG	75	17/11/2008	204	222	22/12/2009	277	400	129.0	120.0	152.0	23.7	33.8
MNCG	75	17/11/2008	204	222	20/02/2010	324	460	134.0	137.0	160.0	22.0	36.5
MNCG	75	17/11/2008	204	222	17/04/2010	371	516	136.0	127.0	125.0	25.0	37.3
JFPA	302	09/11/2008	254	230	05/09/2009	245	300	135.0	124.0	147.0	20.5	
JFPA	302	09/11/2008	254	230	31/10/2009	271	356	139.5	122.0	153.0	23.0	28.5
JFPA	302	09/11/2008	254	230	22/12/2009	314	408	136.0	125.0	160.0	25.5	32.1
JFPA	302	09/11/2008	254	230	20/02/2010	355	468	142.0	148.0	160.0	28.0	33.8
JFPA	302	09/11/2008	254	230	17/04/2010	400	524	145.0	137.0	168.0	32.0	38.5
MVB	911	18/11/2008	228	221	05/09/2009	242	291	129.0	124.0	148.0	22.0	
MVB	911	18/11/2008	228	221	31/10/2009	269	347	136.5	128.0	157.0	22.0	28.4
MVB	911	18/11/2008	228	221	22/12/2009	316	399	135.5	127.0	170.0	25.4	33.9
MVB	911	18/11/2008	228	221	20/02/2010	355	459	136.5	139.0	167.5	27.0	33.6
MVB	911	18/11/2008	228	221	17/04/2010	419	515	140.0	131.0	177.5	34.0	36.4
MVB	905	01/11/2008	224	238	05/09/2009	237	308	126.0	119.0	150.0	21.0	
MVB	905	01/11/2008	224	238	31/10/2009	267	364	124.0	122.0	152.0	22.0	31.1
MVB	905	01/11/2008	224	238	22/12/2009	335	416	132.0	127.0	163.0	26.0	34.2
MVB	905	01/11/2008	224	238	20/02/2010	372	476	133.5	135.5	172.0	32.0	39.3
MVB	905	01/11/2008	224	238	17/04/2010	405	532	141.0	129.0	175.0	33.0	39.3
GZR	99	01/11/2008	230	238	05/09/2009	259	308	131.0	121.0	149.0	20.0	
GZR	99	01/11/2008	230	238	31/10/2009	269	364	138.0	132.0	148.0	21.0	31.0
GZR	99	01/11/2008	230	238	22/12/2009	324	416	137.0	136.0	158.0	24.1	33.6
GZR	99	01/11/2008	230	238	20/02/2010	378	476	141.0	144.5	170.0	25.0	34.6
GZR	99	01/11/2008	230	238	17/04/2010	431	532	142.0	140.0	180.0	29.5	35.9
GZR	100	03/11/2008	227	236	05/09/2009	246	306	137.0	122.0	153.0	19.0	
GZR	100	03/11/2008	227	236	31/10/2009	272	362	137.0	122.0	153.0	20.0	29.5
GZR	100	03/11/2008	227	236	22/12/2009	329	414	139.0	129.0	162.0	23.5	29.8
GZR	100	03/11/2008	227	236	20/02/2010	381	474	145.0	136.0	171.0	23.0	33.0
GZR	100	03/11/2008	227	236	17/04/2010	436	530	147.0	141.5	174.5	29.5	36.2
GZR	101	08/11/2008	205	231	05/09/2009	219	301	131.0	115.0	147.0	19.0	
GZR	101	08/11/2008	205	231	31/10/2009	223	357	133.0	125.0	144.0	19.5	25.4
GZR	101	08/11/2008	205	231	22/12/2009	263	409	137.0	128.0	152.0	22.0	28.4
GZR	101	08/11/2008	205	231	20/02/2010	321	469	138.5	131.0	166.0	24.0	31.7
GZR	101	08/11/2008	205	231	17/04/2010	352	525	139.0	127.0	168.0	29.0	34.1
IVAG	661	05/11/2008	193	234	05/09/2009	204	304	132.0	122.0	140.0	18.5	
IVAG	661	05/11/2008	193	234	31/10/2009	220	360	133.0	123.0	141.0	19.0	26.6
IVAG	661	05/11/2008	193	234	22/12/2009	255	412	136.0	126.0	146.0	20.8	29.0
IVAG	661	05/11/2008	193	234	20/02/2010	302	472	141.0	127.5	160.0	20.5	31.6
IVAG	661	06/11/2008	193	234	17/04/2010	334	527	145.0	128.5	160.0	24.0	32.6
IVAG	669	08/11/2008	231	231	05/09/2009	231	301	135.0	125.0	152.0	21.0	
IVAG	669	08/11/2008	231	231	31/10/2009	250	357	142.5	127.0	148.0	22.0	26.6
IVAG	669	08/11/2008	231	231	22/12/2009	296	409	134.5	135.0	154.0	24.5	30.3
IVAG	669	08/11/2008	231	231	20/02/2010	334	469	139.0	139.0	164.0	27.0	32.0
IVAG	669	08/11/2008	231	231	17/04/2010	383	525	143.0	141.0	167.0	31.5	35.8
IVAG	678	09/11/2008	223	230	05/09/2009	235	300	132.0	119.0	149.0	18.0	
IVAG	678	09/11/2008	223	230	31/10/2009	252	356	139.0	115.0	150.0	22.0	28.1
IVAG	678	09/11/2008	223	230	22/12/2009	306	408	140.0	132.0	157.0	21.6	31.3
IVAG	678	09/11/2008	223	230	20/02/2010	350	468	143.5	134.0	167.0	24.0	33.0
IVAG	678	09/11/2008	223	230	17/04/2010	392	524	146.5	131.0	172.0	29.0	35.0
IVAG	682	10/11/2008	217	229	05/09/2009	232	299	129.0	125.0	150.0	18.5	
IVAG	682	10/11/2008	217	229	31/10/2009	255	355	138.0	124.0	152.0	21.0	30.8
IVAG	682	10/11/2008	217	229	22/12/2009	307	407	139.5	122.0	163.0	24.0	34.1
IVAG	682	10/11/2008	217	229	20/02/2010	335	467	142.5	138.0	168.5	24.5	33.7
IVAG	682	10/11/2008	217	229	17/04/2010	381	523	142.5	129.0	169.0	28.0	35.7
IVAG	694	11/11/2008	219	228	05/09/2009	232	298	134.0	115.5	147.0	20.5	

IVAG	694	11/11/2008	219	228	31/10/2009	241	354	145.5	115.0	152.0	20.0	26.6
IVAG	694	11/11/2008	219	228	22/12/2009	292	406	142.0	123.0	160.0	23.5	31.3
IVAG	694	11/11/2008	219	228	20/02/2010	343	466	147.5	140.0	170.0	24.0	31.8
IVAG	694	11/11/2008	219	228	17/04/2010	391	522	147.5	128.0	173.0	29.0	43.1
IVAG	695	11/11/2008	229	228	05/09/2009	237	298	132.0	127.0	150.0	20.5	
IVAG	695	11/11/2008	229	228	31/10/2009	270	354	138.0	126.0	147.0	23.0	27.8
IVAG	695	11/11/2008	229	228	22/12/2009	309	406	135.5	133.0	154.0	25.5	31.9
IVAG	695	11/11/2008	229	228	20/02/2010	348	466	142.0	140.0	163.5	25.0	33.7
IVAG	695	11/11/2008	229	228	17/04/2010	404	522	144.0	127.0	172.0	28.5	36.5
IVAG	716	16/11/2008	202	223	05/09/2009	223	293	131.0	124.0	144.0	19.5	
IVAG	716	16/11/2008	202	223	31/10/2009	233	349	138.5	124.0	145.0	20.2	29.2
IVAG	716	16/11/2008	202	223	22/12/2009	273	401	137.0	130.0	152.0	25.0	33.4
IVAG	716	16/11/2008	202	223	20/02/2010	327	461	139.5	139.0	159.0	24.0	33.0
IVAG	716	16/11/2008	202	223	17/04/2010	376	517	143.0	142.0	166.0	29.0	34.7
IVAG	717	16/11/2008	192	223	05/09/2009	206	293	130.0	120.0	140.0	19.0	
IVAG	717	16/11/2008	192	223	31/10/2009	202	349	129.5	117.0	144.0	18.5	24.1
IVAG	717	16/11/2008	192	223	22/12/2009	240	401	137.5	122.0	148.0	20.5	27.1
IVAG	717	16/11/2008	192	223	20/02/2010	261	461	140.0	119.0	151.0	19.0	29.1
IVAG	717	16/11/2008	192	223	17/04/2010	300	517	143.0	130.0	152.0	22.5	30.6
IVAG	725	20/11/2008	251	219	05/09/2009	261	289	135.0	126.0	150.0	22.0	
IVAG	725	20/11/2008	251	219	31/10/2009	284	345	134.0	125.0	150.0	26.0	28.1
IVAG	725	20/11/2008	251	219	22/12/2009	345	397	140.5	131.0	165.0	27.5	33.8
IVAG	725	20/11/2008	251	219	20/02/2010	392	457	143.0	145.0	171.0	29.0	34.6
IVAG	725	20/11/2008	251	219	17/04/2010	444	513	148.0	145.0	177.0	33.5	35.4

**Region**	Region from which the animal comes
**Prod.**	Indentification of producer (animal)
**B.Date**	Date of birth
**In.weight**	Weight at start of performance test (kg)
**In. age**	Age at start of test (days)
**Weight date**	Weighing dates
**Weight**	Weight on different weighing dates (kg)
**Age**	Age at each weighing (days)
**HH**	hip height (cm)
**BL**	body length (cm)
**CC**	Chester circunference (cm)
**SC**	Scrotal circufenrence (cm)
**REA**	Rib eye area (cm^2)
